# Mutational signature and clonal relatedness of recurrent urothelial carcinomas with aristolochic acid

**DOI:** 10.3389/fonc.2022.990023

**Published:** 2022-09-14

**Authors:** Jie Zhu, Qing Ai, Qiang Cheng, Dan Shen, Zhouhuan Dong, Jie Li, Donglai Shen, Wei Wang, Xu Zhang, Hongzhao Li

**Affiliations:** ^1^ Senior Department of Urology, The Third Medical Center of Chinese People's Liberation Army General Hospital, Beijing, China; ^2^ Department of Pathology, The First Medical Center of Chinese People's Liberation Army General Hospital, Beijing, China

**Keywords:** urothelial carcinomas, aristolochic acid, mutational signature, tumor microenvironment, clonal relatedness

## Abstract

Urothelial carcinomas (UCs) are malignant tumors that arise from the lower and upper urinary tract and are characterized by multiple recurrences. Aristolochic acid (AA) is a potent nephrotoxin and human carcinogen associated with UC. East Asian populations with a high UC prevalence have an unusual genome-wide AA-induced mutational pattern. To address the genomic differences and clonal relatedness between primary and recurrent tumors in the UCs with AA pattern, we investigated the genomic differences and tumor microenvironment (TME) of AA and non-AA UCs. 17 UC patients were recruited, with nine documented AA exposure. Eleven of them showed recurrence. After-surgery tissues of primary and paired recurrent tumors were collected. Capture-based targeted deep sequencing was performed using a commercial panel consisting of 520 cancer-related genes. Tumor-infiltrating lymphocytes (TILs) were identified with an immunofluorescence-based microenvironment analysis panel (MAP). Hierarchical clustering based on the COSMIC signatures confirmed two significant subtypes: AA Sig and non-AA Sig. AA Sig was associated with AA-containing herbal drug intake, recurrence, and higher tumor mutation burden (TMB). The clonal architecture of UCs revealed three types of clonal evolution patterns. Non-AA Sig cohort showed shared clonal origin of primary and recurrent tumors. AA Sig showed heterogeneity and had multiple independent origins. Recurrent tumors as second primary tumors in AA Sig showed immunoreactive TME, indicating a better response with immune checkpoint inhibitor therapy. The AA mutational signature and unique immune profiles are helpful molecular markers to distinguish AA exposure from other carcinogens. These results also provide new insights into the origin of recurrent UCs that could affect treatment strategies.

## Introduction

Urothelial carcinomas (UCs) are fourth malignant tumors that arise from the upper and lower urinary tract and are characterized by multiple recurrences (1). The majority of patients with UC have more than one tumor in their lifetime. The upper tract urothelial carcinoma (UTUC), which involves the pelvis and ureter, is uncommon and accounts for 5-10% of UC (2). The lower tract urothelial carcinoma (LTUC) is mainly located in the bladder. Despite the low incidence, UTUC is more malignant than LTUC. It is reported that two-thirds of UTUCs are invasive at diagnosis compared with 15-25% of UC of the bladder (3). Recurrence in the bladder occurs in 22-47% of UTUC patients (4). Exposure to environmental carcinogens has long been known to increase the risk of UC and tumor recurrence (5–7). It has been reported that the consumption of Chinese herbs that contain aristolochic acid (AA) has been associated with an increased risk of urinary tract cancers (8–11).

AA is a potent carcinogen derived from Aristolochia plants that are used in traditional herbal medicine. AA exposure can induce an unusual genome-wide AA mutational signature characterized by A:T to T:A transversions, a relatively unusual type of mutation infrequently seen in many types of cancer (12–14). This AA signature, termed signature 22 in COSMIC, holds great potential as a “molecular fingerprint” for AA exposure in UTUC (14). Besides UC, AA signature is also detected in multiple cancer types, such as renal cell carcinomas, clear cell renal cell carcinoma, and hepatocellular carcinomas (12, 15, 16). However, the association of AA with recurrent UC remains largely unexplored.

Two theories have been proposed to explain the clonal relatedness of UC recurrence. One theory suggests that recurrent tumors share the same genomic characterization as primary UC tumors. The second theory indicates recurrent tumors may arise as second primary tumors causing independent genetic alterations at different sites. However, the study of clonal relatedness of UC recurrence with AA exposure is very limited.

We performed next-generation sequencing (NGS) on tumor specimens of 17 Chinese UC patients with or without AA exposure, including the recurrent tumors. Using the AA signature as a screening tool, we could identify the AA cohort and non-AA cohort and explore the potential clinical utility of tumor genomic characterization in patients of the AA cohort. We were interested in addressing the genomic differences between primary and recurrent tumors in the AA cohort to determine whether clinical characteristics reflect differences in genomic characterization. In particular, we sought to address whether single or multiple recurrent tumors in individual patients are clonally related recurrences or represent distinct primary tumors. Furthermore, we also characterized the tumor microenvironment (TME) of AA UCs.

## Materials and methods

### Patient and study design

A total of 17 patients diagnosed with urothelial carcinomas were retrospectively analyzed in our study. Resection was performed for each tumor lesion at the PLA General Hospital. None of them underwent chemotherapy or radiation therapy. Of the 17 patients, 11 (p1-p11) had metachronous recurrences with an interval time of more than three months. After-surgery tissues of primary and paired recurrent tumors were collected for NGS and immunofluorescence-based microenvironment analysis. Clinical data were obtained, including the patient’s age, sex, AA- intake history, body mass index (BMI), concomitant diseases, tumor size, pathological stage, tumor sites, and recurrence-free survival (RFS) from the electronic medical health records. This study was approved by the Ethics Committee of the PLA General Hospital. Written informed consent was obtained from each patient.

### AA Exposure assessment

AA exposure assessment was defined according to previous research assessed by self-reported data (17, 18): (1) the presence of a definite history of taking AA-containing herbal drug, Guan Mu Tong (*Aristolochia manshuriensis*), Guang Fangchi (*Aristolochia fangchi*), Qing Mu Xiang (*Radix Aristolochiae*), Ma Dou Ling (*Fructus Aristolochiae*), Tian Xian Teng (*Caulis Aristolochiae*), Xun Gu Feng (*herba Aristolochiae mollissimae*), and Zhu Sha Lian (*Kaempfer Dutchmanspipe Root*); (2) the duration of exposure of the above drugs was more than three months.

### Sequencing analysis

Genomic profiling was performed using a panel covering 520 cancer-related genes (OncoScreen plus, Burning Rock Biotech, Guangzhou, China), allowing for the evaluation of complex biomarkers such as tumor mutational burden (TMB). The Catalogue of Somatic Mutations in Cancer (COSMIC) mutational signatures were downloaded from the COSMIC website (https://cancer.sanger.ac.uk/cosmic).

### Microenvironment analysis

Tumor tissues were used to perform immunofluorescence-based tissue microenvironment analysis. Multiplex staining for seven key immune markers, PD1, PD-L1, CD3, CD8, CD56, CD 68, and CD163, was performed with PANO 7-plex IHC kit (Panovue, Beijing, China) according to the manufacturer’s protocol. All stained tissues were independently scored by two pathologists who were blinded to the clinical parameters. Our study reported the density and positive rate of tumor-infiltrating lymphocytes (TILs) in both parenchymal (iTILs) and stromal (sTILs) compartments.

### Statistical analyses

Statistical analysis was performed using R version 4.1.0. The Fisher exact test or a non‐parametric test was used to compare categorical data. The Wilcoxon test between groups analyzed the difference in the infiltration of immune markers and TMB. P-values <0.05 were considered statistically significant.

## Results

### AA UC-specific DNA mutational signatures

Targeted NGS was conducted across multiple tumor specimens in 17 patients, 9 of whom have documented AA exposure. At diagnosis, the median age was 67.2, and the median primary tumor size was 3.1 cm. Most patients were women (n = 10). No Obese (BMI > 30 kg/m^2^) was identified in all the patients. Most patients were overweight (BMI 25.1–30 kg/m^2^) and normal weight (BMI 18.5–25 kg/m^2^). Nine had at least one concomitant disease, including aristolochic acid nephropathy, chronic kidney failure, coronary artery disease, diabetes, hypertension, and renal failure. Fourteen patients had UC originating from the upper tract, and the remainder had LTUC of the bladder (n = 3). The majority of tumor stage was T2 or worse, and none of them had shown metastasis. 82.4% of tumors were high-grade. Patient demographics and clinical features were provided in **Table 1**.

**Table 1:** Patient characteristics.

	Overall (n = 17)	AA (n = 11)	non-AA (n = 6)
Age (median)	63.2	63	63.7
Gender			
F	10	7	3
M	7	4	3
AA intake			
Yes	9	9	0
No	8	2	6
BMI (kg/m²)			
< 18.5	1	1	0
18.5 - 24.9	5	4	1
25.0 - 29.9	5	4	1
> 29.9	0	0	0
Unknown	6	2	4
Number of concomitant diseases			
0	2	0	2
1	5	5	0
2	3	3	0
3	1	1	0
Unknown	6	2	4
Tumor size (cm)	3.1	3.25	2.5
Primary tumor site			
Upper tract	14	8	6
Lower tract	3	3	0
Tumor stage			
Ta	2	2	0
T1	4	4	0
≥T2	10	4	6
Unknown	1	1	0
Grade			
Low-grade	2	2	0
High-grade	14	8	6
Unknown	1	1	0
Recurrence			
Yes	11	9	2
No	6	2	4

AA, aristolochic acid; BMI, body mass index; Concomitant diseases include aristolochic acid nephropathy, chronic kidney failure, coronary artery disease, diabetes, hypertension, and renal failure.

**Figure 1:**
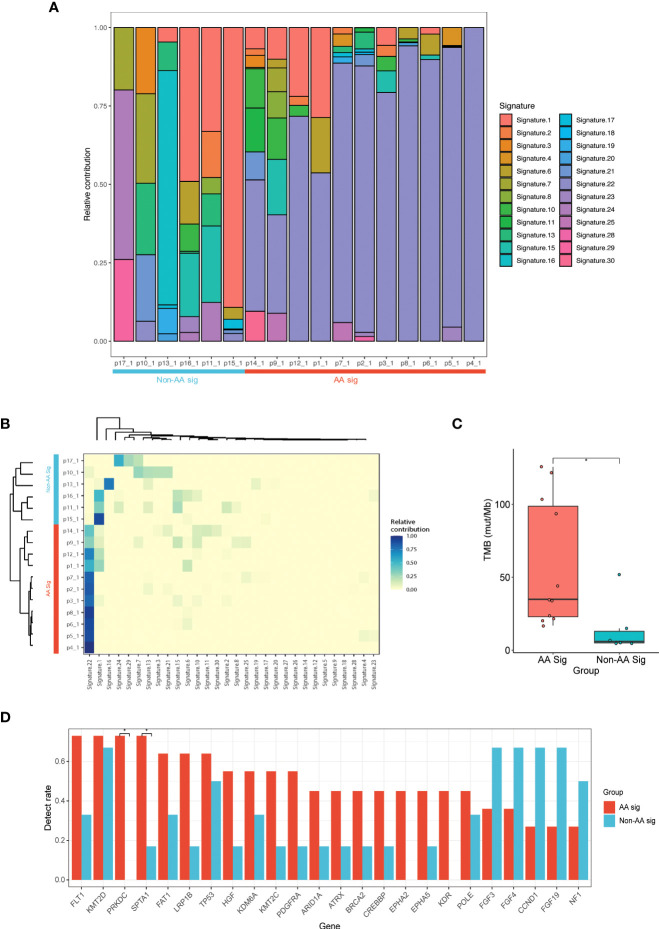
NGS of UC patients with or without AA signature. **(A)** Landscape of mutation signatures of AA UC tumors and non-AA UC tumors. Patient primary UC samples were labeled as p1_1 - p17_1. **(B)** Hierarchical clustering of mutation signatures revealing AA signature (AA Sig) and non-AA signature (Non-AA Sig) group. **(C)** Bar plot of TMB representing median TMB for AA UC tumors versus non-AA UC tumors. **(D)** The detection rate of the top 24 mutated genes in all patients. *p < 0.05; NGS, next-generation sequencing; AA, aristolochic acid; UC, urothelial carcinoma; TMB, tumor mutational burden.

Next, we sought to determine whether UC showed specific mutational signatures with AA exposure. Using the COSMIC, SBS V2 signatures of mutational processes in human cancer, we identified 24 mutational signatures (**Figure 1A**). Hierarchical clustering based on the COSMIC signatures confirmed two significant cohorts: AA Sig (n=11) and non-AA Sig (n=6) (**Figure 1B**). There was a significantly higher number of signature 22 mutations in the AA Sig cohort than in the non-AA Sig cohort. Analysis showed that the AA Sig cohort was associated with AA-containing herbal drug intake, recurrence (**Table 1**), and higher TMB (35 vs. 6 mut/Mb, **Figure 1C**). The AA mutational signature was observed in two patients’ tumors without previous indication of AA exposure (**Table 1**).

We identified 864 mutations in the two cohorts, with a median of 38 (range, 19 to 137) somatic mutations per tumor in AA UC patients versus 18 (range, 11 to 69) in non-AA. 276 and 102 genes were mutated in the AA Sig cohort and non-AA Sig cohort in total, making it difficult to distinguish mutated drivers from mutated passenger genes. The most frequently mutated genes in AA Sig and non-AA Sig included *KMT2D* (72.7% and 66.6%), *TP53* (63.6% and 50%), and *FLT1* (72.7% and 33.3%, **Figure 1D**). The mutation frequency of *FLT1*, *SPTA1* and *PRKDC* was higher in AA Sig patients than non-AA Sig (72.7% vs. 33.3%, 72.7% vs. 16.7%, and 72.7% vs. 0%, respectively; p < 0.05). *FGF3* was higher in non-AA Sig patients (36.3% vs. 66.7%).

**Figure 2:**
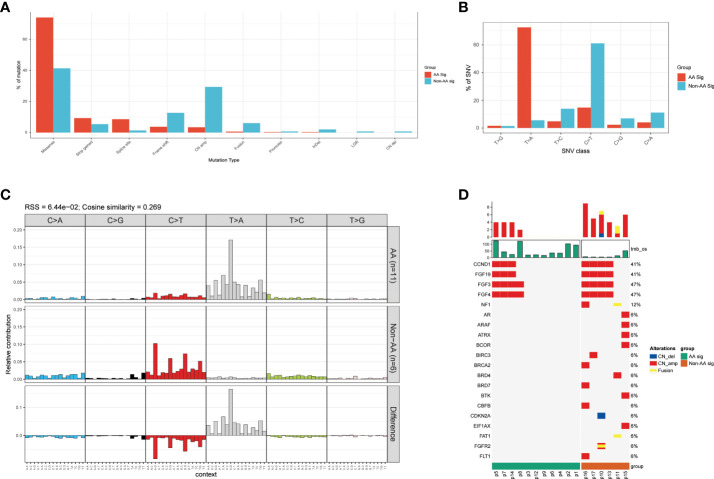
Mutational pattern in AA Sig and non-AA Sig cohorts **(A)** The detection rates of different mutation types in both cohorts. **(B)** percentage of SNV mutations in each of six possible mutation classes in both cohorts. **(C)** Trinucleotide contexts for SNVs in each of six possible mutation classes in both cohorts. **(D)** Copy number variations of the two cohorts. SNV, single nucleotide variation.

The vast majority (74.1% and 41.3% in AA Sig and non-AA Sig cohorts, respectively, **Figure 2A**) of these mutations were single-nucleotide variation (SNV) missense mutations. The SNVs in the AA Sig showed a marked mutagenic signature, with T>A missense accounting for 72.6% of SNVs. In contrast, C>T missense accounted for 61.1% in the non-AA cohort (**Figure 2B**). In AA Sig, we also observed a preference for a C in the base preceding the mutated T residue and a preference for G at the following base in T>A mutation (**Figure 2C**). We also found copy number variations (CNVs) in both cohorts, and the frequency of CNVs in non-AA Sig was higher (**Figure 2D**).

### Genomic differences between primary and recurrent tumors

**Figure 3:**
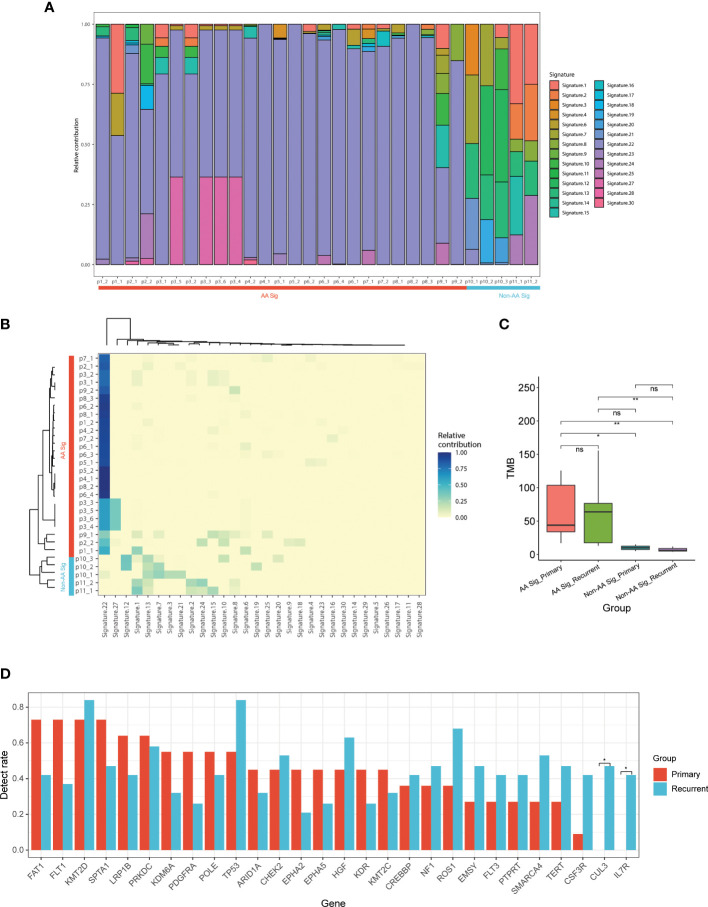
Mutational signature of primary and recurrent tumors in AA UC and non-AA UC cohorts **(A)** Landscape of mutation signatures of all the primary and recurrent tumor samples. **(B)** Hierarchical clustering of mutation signatures in all samples. **(C)** Bar plot of TMB representing median TMB for primary and recurrent tumors in AA UC and non-AA UC cohorts. **(D)** The detection rate of the top 28 mutated genes in all patients. *p < 0.05; **p < 0.01; ns, not significant.

To delineate differences between recurrent AA Sig and non-AA Sig, we compared the mutational signatures of 30 tumor specimens from 11 recurrent UC patients, in which 4 of them developed multiple recurrences (p3, 6, 8, and p10) (**Figure 3A**). 9 out of the 11 recurrent patients identified AA signature and the recurrent tumors in the AA Sig cohort consistently showed AA signature, which indicated that AA exposure might contribute to recurrences (**Figure 3B**). In both cohorts, the TMB in recurrent tumors was similar to primary tumors (**Figure 3C**). The most frequently mutated genes in primary and recurrent tumors included *KMT2D* (72.7% and 84.2%), *TP53* (54.5% and 84.2%), *FAT1* (72.7% and 42.1%), and *FLT1* (72.7% and 42.1%, **Figure 3D**). These results demonstrated the mutation signature between primary and recurrent tumors was similar. The AA mutational signature was consistently identified in paired tumors of AA UC cohort but not non-AA cohort.

### Clonal relatedness of primary and recurrent tumors in AA UC and non-AA UC patients

**Figure 4:**
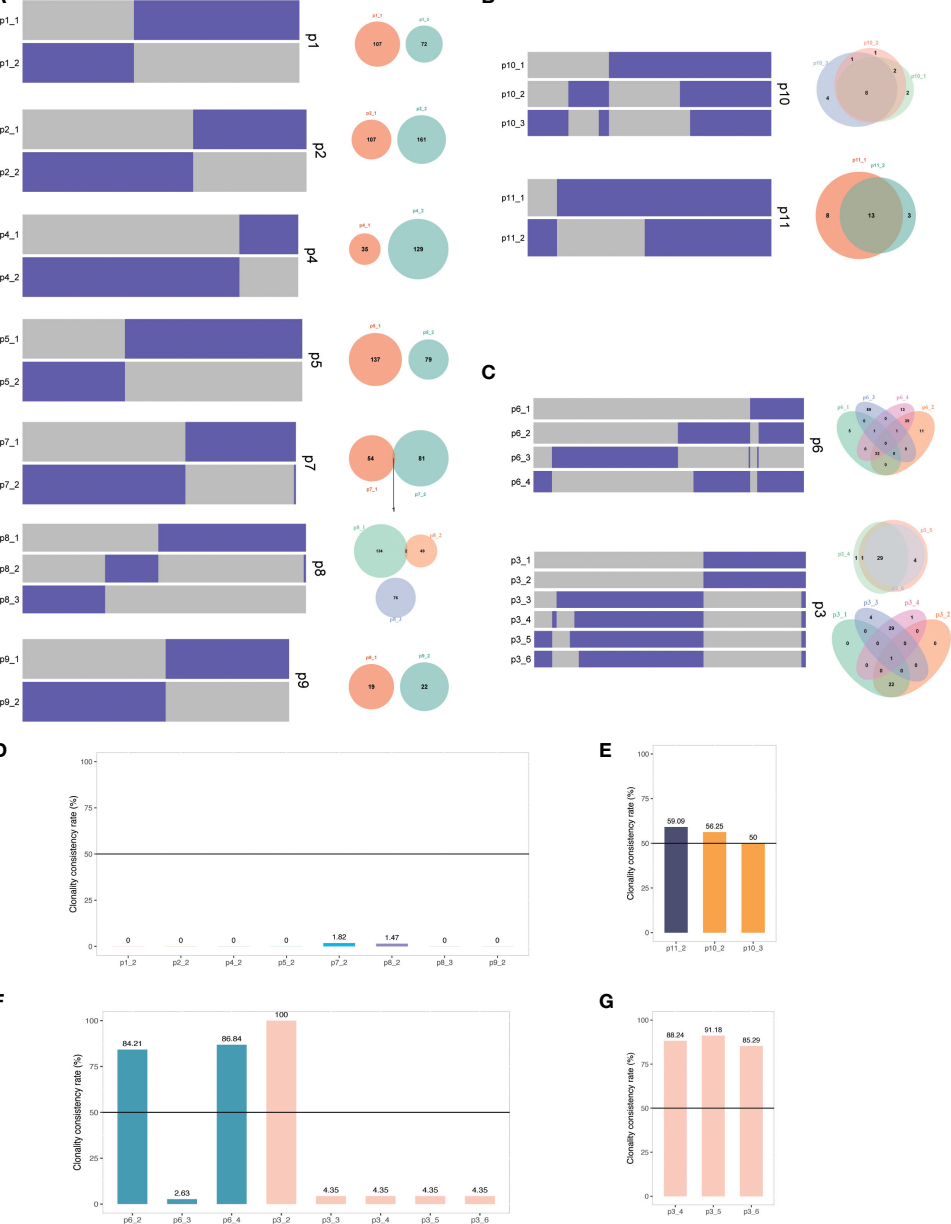
The clonal relatedness of primary and recurrent tumors **(A)** Heat maps show the presence (purple) or absence (grey) of mutations in primary and recurrent tumors of p1, 2, 4, 5, 7, 8, and 9. Venn diagrams present the total number of mutations unique or shared between primary and recurrent tumors. **(B)** Heat maps and Venn diagrams of p10 and p11. **(C)** Heat maps and Venn diagrams of p3 and p6. **(D)** Clonality consistency rate of recurrent tumors compared with the primary tumors. **(E)** Clonality consistency rate of p10 and p11. **(F)** Clonality consistency rate of p3 and p6. **(G)** Clonality consistency rate of p3 recurrent tumors compared with the third tumor (50%). Black lines indicate the cutoff value to define clonal relatedness.

The clonal evolution theory of UC, especially the UC with AA signature, is not well studied. The single or multiple recurrent tumors arising in individual patients are clonally related recurrences if they inherit an identical or similar set of somatic genetic alterations; otherwise, they may represent distinct primary tumors. To further determine the clonal relatedness of the recurrent tumors, we probed the genetic aberrations identified by NGS of all primary and recurrent tumors, including multiple recurrences in patients 3, 6, 8, and 10. Of the 11 patients (p1-p11), nine were from the AA Sig cohort (p1-p9) and two were from the non-AA Sig cohort (p10, p11). Our results revealed three types of clonal evolution patterns in our patients (**Figure 4**). Clonal architecture of UCs indicated that the paired recurrent tumors in 7 patients (63.6%) shared minor genetic alterations with the primary tumors, revealing their different clonal origins (**Figure 4A**). Less than 2% of somatic mutations were present in primary and recurrent tumors (Fig 4D). These recurrent cases were considered second primary tumors since the primary and recurrent tumors arose from independent clones. Of note, they were all classified as AA Sig cohort. However, in the non-AA Sig cohort, shared mutations were found in primary tumors and recurrent tumors (**Figure 4B**). The shared mutations accounted for 59.09% of all the mutations in p11, and 56.25% and 50% of all the mutations in p12 who developed recurrences twice (**Figure 4E**), demonstrating shared clonal origin of primary and recurrent tumors.

In addition, the clonal origins of some patients showed heterogeneity (**Figure 4C**). When compared with the primary tumor of p6, 84.21% and 86.84% of shared mutations were present in the second (p6-2) and fourth (p6-4) tumor, but only 2.63% were found in the third tumor (p6-3), which was considered second primary tumor (**Figure 4F**). In another patient, p3, the second tumor inherited an identical set of somatic genetic alterations from the primary tumor (**Figure 4F**). However, the third tumor only shared 4.35% somatic genetic alterations with the primary tumor. It was considered the second primary tumor, with which more than 85% of mutations of the later recurrences were shared (**Figure 4G**). These data suggest that these AA UCs likely have multiple independent origins.

### Tumor microenvironment of recurrent tumors

To characterize the TME of UCs, we compared the density and positive cell rate of seven key immune markers, PD1, PD-L1, CD3, CD8, CD56, CD68, and CD163, with multiplex staining. In primary tumors, there was no significant difference between AA Sig and non-AA Sig patients (**Figure S1A**). We further compared the expression of each marker in sTILs and iTILs, and AA UC patients had significantly higher CD56+ iTILs (**Figure S1B**).

**Figure 5:**
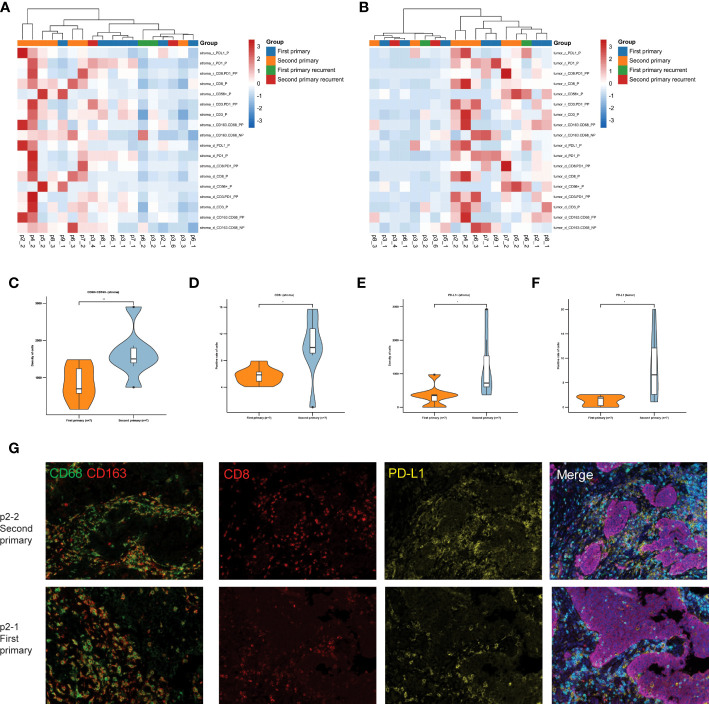
TME analysis of tumors with similar and different clonal origins. **(A)** The heat map shows the density and positive cell rate of seven key immune markers, PD1, PD-L1, CD3, CD8, CD56, CD 68, and CD163, in the stroma of patients with recurrence. **(B)** The heat map shows the density and positive cell rate of seven key immune markers in tumor of patients with recurrence. **(C)** The density of CD68 + CD163- sTILs in first primary group and second primary group. **(D)** The positive cell rate of CD8+ sTILs. The density of PD-L1+ sTILs **(E)** and iTILs **(F)** in first primary group and second primary group. **(G)** Representative immunofluorescence micrographs of primary tumors (lower panels) and second primary tumors (upper panels) in p2. Double staining of CD68 (green) and CD 163 (red) shown on the left panels. Merged image of CD8 (red), PD-L1 (yellow), PD1 (green), PD-L1 (yellow), CD3 (indigo), pan-Cytokeratin (purple) and DAPI (blue) shown on the right panels. Original magnification ×100. *, p ≤ 0.05; **, p ≤ 0.01.

In particular, we analyzed the expression of these immune markers between primary and recurrent tumors based on their clonal relatedness. The primary tumors of each patient were classified as group “First primary.” The recurrent tumors were classified as group “First primary recurrent,” in which shared mutations were detected in the recurrent tumors in p6-2, p6-4, and p3-2. Little or no common mutations of primary tumors were observed in the recurrent tumors in p1-2, p2-2, p4-2, p5-2, p7-2, p8-2, p9-2, p3-3, and p6-3 (group “Second primary”), and their clonally related recurrent tumors p3-4/5/6 were classified as group “Second primary Recurrent.” The TME of second primary tumors showed different TME between first primary and recurrent tumors in tumor nests and stroma (**Figures 5A, B**). With paired samples Wilcoxon test, second primary tumors were associated with significantly higher CD68+CD163- sTILs (marker of M1 macrophages, **Figure 5C**), CD8+ cytotoxic sTILs (**Figure 5D**), PD-L1+ sTILs (**Figure 5E**) and iTILs (**Figure 5F**). These data indicated the immunoreactive TME (**Figure 5G**) in second primary tumors.

**Figure 6:**
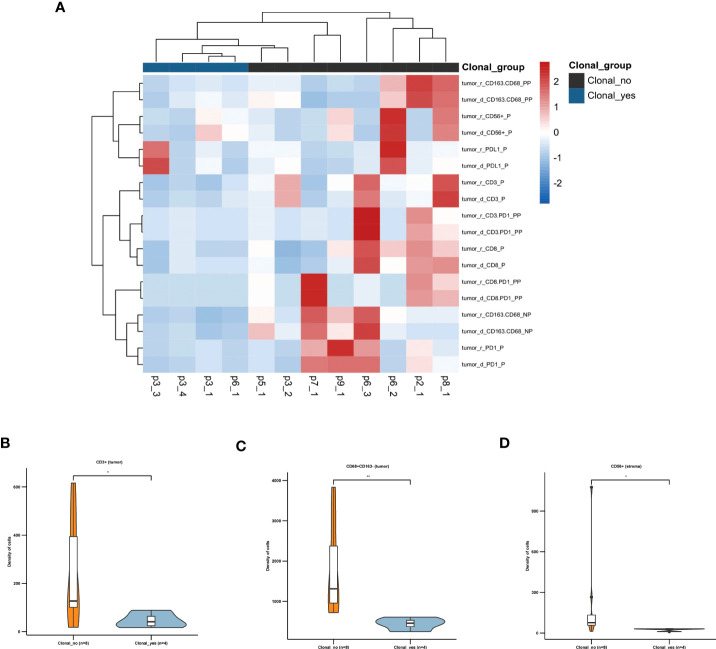
TME is associated with the pattern of clonal origin of recurrent tumors **(A)** The heat map shows the density and positive cell rate of seven key immune markers, PD1, PD-L1, CD3, CD8, CD56, CD 68, and CD163, in tumors of patients with recurrence. **(B)** The density of CD3+ iTILs. **(C)** The density of CD68 + CD163- sTILs. **(D)** The density of CD56+ iTILs. *, p ≤ 0.05; **, p ≤ 0.01.

To investigate whether the TME plays a role in the development of recurrent tumors, we classified all the tumor specimens into two groups based on their clonal relatedness compared with the tumor before recurrence, similar clonal (clonal-yes: p3-1, p3-3, p3-4, p6-1) and different clonal origin (clonal-no: p1-1, p2-1, p4-1, p5-1, p7-1, p8-1, p9-1, p3-2, p6-2, p6-3). Microenvironment analysis showed that the clonal-yes group had fewer iTILs than the clonal-no group (**Figure 6A**). We found that CD3+ cytotoxic iTILs, CD68+CD163- sTILs, CD56+ iTILs were highly infiltrated in recurrent tumors with different clonal origins (**Figures 6B–D**).

These results suggested the primary AA UC tumors with immunoreactive TME constituted of CD3+ T cells, M1 macrophages, and natural killer (NK) may develop recurrence with new clonal origin.

## Discussion

Tumor genomic profiling can provide information that physicians could use to identify potential therapeutic targets and thus guide treatment selection (19). We have presented AA-specific mutational signature in 17 UC patients with 82% (9/11) of AA signature cohort treated with AA. Although the clinical characteristics were similar between AA and non-AA UC, significant differences in genomic alterations were observed. Our analysis showed that AA UC was associated with AA-containing herbal drug intake, recurrence, and higher TMB. There were more mutated genes in AA UC compared with non-AA UC. The most frequently mutated genes in AA UC and non-AA UC were *KMT2D*, and *TP53*. The mutation frequency of *FLT1*, *SPTA1*, and *PRKDC* was higher in AA UC patients than in non-AA UC, but *FGF3* was lower.

A meta-analysis showed that BMI in patients with UC after surgery was a prognostic marker, overweight was a protective factor, while obesity and underweight predicted unfavorable survival (20). Our study showed no significant difference in BMI between the AA and non-AA groups. No patient was obese, and most patients were normal and overweight. The BMI may not contribute to the genomic difference in this study. We also found patients in the AA group had a higher number of concomitant diseases. It has been reported AA was associated with aristolochic acid nephropathy(AAN), chronic kidney disease, and impaired renal function (21–23), which were also observed in our patients. A previous study has identified a strong time-dependent correlation between the duration of AA exposure and the risk of urothelial carcinoma recurrence (17). Therefore, we should do our best to prevent further exposure to AA-containing herbs.

To our surprise, the clonal relatedness of primary and recurrent tumors in the AA UC and non-AA UC cohorts differed; even more, patients in the AA UC cohort showed heterogeneity. In most (7/9) of recurrent patients in the AA UC cohort, the paired recurrent tumors shared no genetic alterations with the primary tumors, revealing their independent origins. The other two UC patients had more than one recurrence, and the mutation pattern between distant recurrences was different. Notably, most AA UC patients with recurrence were found to be independent origins of recurrent tumors, which indicated the recurrent tumors might be secondary primary tumors. Furthermore, the initial primary UC and subsequent recurrence shared most mutations, suggesting clonal similarity without AA exposure. Similar results were reported in recurrent UTUC and UC of the bladder, which observed lesion-to-lesion heterogeneity with 69-86% of mutations shared between the initial primary UC and subsequent recurrent UTUC (24).

Although comparing UTUC and LTUC was not the focus of this study, three patients in the AA cohort were diagnosed with UC of the bladder, and the analysis here showed that the mutation patterns of UC of the bladder were very similar to UTUC with the AA signature, which was consistent with the previous report (25).

Immunotherapy has been developed and clinically applied to manage UC, including bladder and upper urinary tract cancers (26–28). Although no commercialized biomarker is available, TMB shows an obvious predictive implication for the response to immune checkpoint inhibitor (ICI). In a trial on atezolizumab in UC, the median mutational load of responders was higher than that of non-responders (12.4 vs. 6.4 mut/Mb) (29). Thanks to recent progress in genetic sequencing, distinct genetic mutations of AA-associated UTUC have been reported. AA-associated UC harbored higher TMB than non-AA UC. It is plausible to test whether AA-derived DNA adducts or the increased mutation load could be implemented as a prognostic biomarker prior to ICI therapy. Even with the high TMB in the AA UC patents, their TME showed heterogeneity between tumors. It’s urgent to explore prognostic predictors and expand our knowledge of the intrinsic subtyping for AA UCs. We found that recurrent tumors as second primary tumors in AA UC showed immunoreactive TME, indicating a better response with ICI therapy.

There are several limitations in our small retrospective study. Due to the low incidence rate of AA-associated UC, the sample size of our study is small, and we can only provide descriptive observations. Besides AA exposure, there are many other risk factors for urothelial carcinomas (2), such as smoking. More clinical characteristics are needed to explore the factors affecting TMB and TME of UCs. Future studies are needed to confirm our conclusions in a larger population. The median follow-up duration was 24 months (3.5 to 109 months). A longer follow-up is necessary to detect the tumor recurrence.

In conclusion, our findings imply that AA is a potential mutagenic factor leading to recurrent UCs with unique AA mutational signature. The clonal relationships of primary and recurrent tumors showed heterogeneity, and most of the recurrent tumors of AA UC were independent second primary tumors. The unique immune profiles help understand clonal relationships of multiple AA recurrent tumors, which could affect treatment strategies of AA UCs.

## Data availability statement

The original contributions presented in the study are included in the article/**Supplementary Material**. Further inquiries can be directed to the corresponding authors.

## Ethics statement

This study was reviewed and approved by Ethics Committee of the PLA General Hospital. The patients/participants provided their written informed consent to participate in this study.

## Author contributions

Study concept and design: HL, XZ. Acquisition of data: JZ, DS, ZD, DLS. Analysis and interpretation of data: JZ, QC, WW, JL. Drafting of the manuscript: All authors. Critical revision of the manuscript for important intellectual content: All authors. Statistical analysis: JZ, QA. Supervision: HL, XZ. All authors contributed to the article and approved the submitted version.

## Funding

Military top-notch talents fund.

## Acknowledgments

We thank Dr. Ying Sun, Dr. Jinlei Song, Dr. Fei Du, Dr. Lan Su, Dr. Danhua Wang, Ms. Chunyan Wang, and Dr. Chunxiao Pan from the Burning Rock Biotech Company for their support.

## Conflict of interest

The authors declare that the research was conducted in the absence of any commercial or financial relationships that could be construed as a potential conflict of interest.

## Publisher’s note

All claims expressed in this article are solely those of the authors and do not necessarily represent those of their affiliated organizations, or those of the publisher, the editors and the reviewers. Any product that may be evaluated in this article, or claim that may be made by its manufacturer, is not guaranteed or endorsed by the publisher.

## References

[1] Siegel RL Miller KD Jemal A. Cancer statistics, 2020. CA Cancer J Clin (2020) 70(1):7–30. doi: 10.3322/caac.21590 31912902

[2] Rouprêt M Babjuk M Burger M Capoun O Cohen D Compérat EM. European Association of urology guidelines on upper urinary tract urothelial carcinoma: 2020 update. Eur Urol (2021) 79(1):62–79. doi: 10.1016/j.eururo.2020.05.042 32593530

[3] Margulis V Shariat SF Matin SF Kamat AM Zigeuner R Kikuchi E. Outcomes of radical nephroureterectomy: A series from the upper tract urothelial carcinoma collaboration. Cancer (2009) 115(6):1224–33. doi: 10.1002/cncr.24135 19156917

[4] Xylinas E Rink M Margulis V Karakiewicz P Novara G Shariat SF. Multifocal carcinoma *in situ* of the upper tract is associated with high risk of bladder cancer recurrence. Eur Urol (2012) 61(5):1069–70. doi: 10.1016/j.eururo.2012.02.042 22402109

[5] Green DA Rink M Xylinas E Matin SF Stenzl A Roupret M. Urothelial carcinoma of the bladder and the upper tract: disparate twins. J Urol (2013) 189(4):1214–21. doi: 10.1016/j.juro.2012.05.079 23023150

[6] Colin P Koenig P Ouzzane A Berthon N Villers A Biserte J. Environmental factors involved in carcinogenesis of urothelial cell carcinomas of the upper urinary tract. BJU Int (2009) 104(10):1436–40. doi: 10.1111/j.1464-410X.2009.08838.x 19689473

[7] Miyazaki J Nishiyama H. Epidemiology of urothelial carcinoma. Int J Urol (2017) 24(10):730–4. doi: 10.1111/iju.13376 28543959

[8] Lai MN Wang SM Chen PC Chen YY Wang JD. Population-based case-control study of Chinese herbal products containing aristolochic acid and urinary tract cancer risk. J Natl Cancer Inst (2010) 102(3):179–86. doi: 10.1093/jnci/djp467 PMC2815723 20026811

[9] Cosyns JP Jadoul M Squifflet JP Wese FX van Ypersele de Strihou C. Urothelial lesions in Chinese-herb nephropathy. Am J Kidney Dis (1999) 33(6):1011–7. doi: 10.1016/S0272-6386(99)70136-8 10352187

[10] Nortier JL Martinez MC Schmeiser HH Arlt VM Bieler CA Petein M. Urothelial carcinoma associated with the use of a Chinese herb (Aristolochia fangchi). N Engl J Med (2000) 342(23):1686–92. doi: 10.1056/NEJM200006083422301 10841870

[11] Arlt VM Stiborova M Schmeiser HH. Aristolochic acid as a probable human cancer hazard in herbal remedies: A review. Mutagenesis. (2002) 17(4):265–77. doi: 10.1093/mutage/17.4.265 12110620

[12] Poon SL Pang ST McPherson JR Yu W Huang KK Guan P. Genome-wide mutational signatures of aristolochic acid and its application as a screening tool. Sci Transl Med (2013) 5(197):197ra01. doi: 10.1126/scitranslmed.3006086 23926199

[13] Moriya M Slade N Brdar B Medverec Z Tomic K Jelaković B. TP53 mutational signature for aristolochic acid: An environmental carcinogen. Int J Cancer (2011) 129(6):1532–6. doi: 10.1002/ijc.26077 21413016

[14] Hoang ML Chen CH Sidorenko VS He J Dickman KG Yun BH. Mutational signature of aristolochic acid exposure as revealed by whole-exome sequencing. Sci Transl Med (2013) 5(197):197ra02. doi: 10.1126/scitranslmed.3006200 PMC3973132 23926200

[15] Jelaković B Castells X Tomić K Ardin M Karanović S Zavadil J. Renal cell carcinomas of chronic kidney disease patients harbor the mutational signature of carcinogenic aristolochic acid. Int J Cancer (2015) 136(12):2967–72. doi: 10.1002/ijc.29338 PMC4720973 25403517

[16] Scelo G Riazalhosseini Y Greger L Letourneau L Gonzàlez-Porta M Wozniak MB. Variation in genomic landscape of clear cell renal cell carcinoma across Europe. Nat Commun (2014) 5:5135. doi: 10.1038/ncomms6135 25351205

[17] Zhong W Zhang L Ma J Shao S Lin R Li X. Impact of aristolochic acid exposure on oncologic outcomes of upper tract urothelial carcinoma after radical nephroureterectomy. Onco Targets Ther (2017) 10:5775–82. doi: 10.2147/OTT.S148641 PMC5722008 29255365

[18] Lu H Liang Y Guan B Shi Y Gong Y Li J. Aristolochic acid mutational signature defines the low-risk subtype in upper tract urothelial carcinoma. Theranostics. (2020) 10(10):4323–33. doi: 10.7150/thno.43251 PMC7150494 32292497

[19] Cheng ML Berger MF Hyman DM Solit DB. Clinical tumour sequencing for precision oncology: time for a universal strategy. Nat Rev Cancer (2018) 18(9):527–8. doi: 10.1038/s41568-018-0043-2 PMC6618305 30030494

[20] Yang Z Bai Y Hu X Wang X Han P. The prognostic value of body mass index in patients with urothelial carcinoma after surgery: A systematic review and meta-analysis. Dose Response (2020) 18(4):1559325820979247. doi: 10.1177/1559325820979247 33402880 PMC7745568

[21] Rebhan K Ertl IE Shariat SF Grollman AP Rosenquist T. Aristolochic acid and its effect on different cancers in uro-oncology. Curr Opin Urol (2020) 30(5):689–95. doi: 10.1097/MOU.0000000000000806 32701724

[22] Shan H Tian W Hong Y Xu B Wang C Yu B. Clinicopathologic characteristics and prognosis of upper tract urothelial carcinoma complicated with aristolochic acid nephropathy after radical nephroureterectomy. BMC Complement Med Ther (2020) 20(1):166. doi: 10.1186/s12906-020-2861-5 32493345 PMC7268428

[23] Dickman KG Chen C-H Grollman AP Pu Y-S. Aristolochic acid-containing Chinese herbal medicine and upper urinary tract urothelial carcinoma in Taiwan: a narrative review. World J Urol (2022). doi: 10.1007/s00345-022-04100-5 35867141

[24] Audenet F Isharwal S Cha EK Donoghue MTA Drill EN Ostrovnaya I. Clonal relatedness and mutational differences between upper tract and bladder urothelial carcinoma. Clin Cancer Res (2019) 25(3):967–76. doi: 10.1158/1078-0432.CCR-18-2039 PMC6359971 30352907

[25] Poon SL Huang MN Choo Y McPherson JR Yu W Heng HL. Mutation signatures implicate aristolochic acid in bladder cancer development. Genome Med (2015) 7(1):38. doi: 10.1186/s13073-015-0161-3 26015808 PMC4443665

[26] Donin NM Lenis AT Holden S Drakaki A Pantuck A Belldegrun A. Immunotherapy for the treatment of urothelial carcinoma. J Urol (2017) 197(1):14–22. doi: 10.1016/j.juro.2016.02.3005 27460757

[27] Chism DD. Urothelial carcinoma of the bladder and the rise of immunotherapy. J Natl Compr Canc Netw (2017) 15(10):1277–84. doi: 10.6004/jnccn.2017.7036 28982752

[28] Kim J. Immune checkpoint blockade therapy for bladder cancer treatment. Investig Clin Urol (2016) 57 Suppl 1(Suppl 1):S98–s105. doi: 10.4111/icu.2016.57.S1.S98 PMC4910761 27326412

[29] Rosenberg JE Hoffman-Censits J Powles T van der Heijden MS Balar AV Necchi A. Atezolizumab in patients with locally advanced and metastatic urothelial carcinoma who have progressed following treatment with platinum-based chemotherapy: a single-arm, multicentre, phase 2 trial. Lancet (2016) 387(10031):1909–20. doi: 10.1016/S0140-6736(16)00561-4 PMC5480242 26952546

