# Supplementary material for: Mutational signature and clonal relatedness of recurrent urothelial carcinomas with aristolochic acid
Source: Front Oncol. 2022 Sep 14;12:990023. doi: 10.3389/fonc.2022.990023 (PMC9516318; doi:10.3389/fonc.2022.990023)
Supplement: Supplementary file 1 [file DataSheet_1.docx]

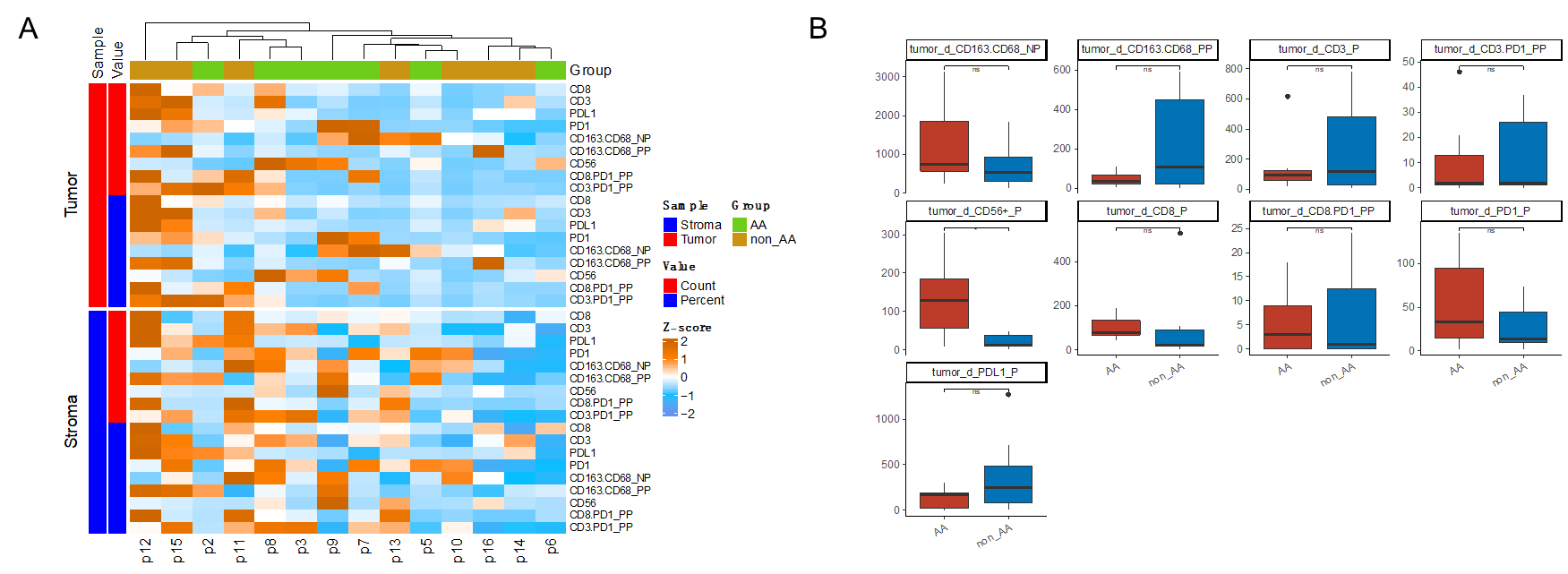


Supplementary Figure 1. Immune microenvironment analysis of tumors in the AA and non-AA cohort.

A. The heat map shows the density and positive cell rate of seven key immune markers, PD1, PD-L1, CD3, CD8, CD56, CD 68, and CD163, in primary tumors of the AA and non-AA cohort. B. Compare of the density of CD163- CD68+, CD163+ CD68+, CD3+, CD3+ PD1+, CD56+, CD8+, CD8+ PD1+, PD1+, PD-L1+ iTILs in AA and non-AA cohort. *, p < 0.05; ns = not significant.
